# Mass Spectrometric Imaging of Plasma Membrane Lipid Alteration Correlated with Amperometrically Measured Activity-Dependent Plasticity in Exocytosis

**DOI:** 10.3390/ijms21249519

**Published:** 2020-12-14

**Authors:** Chaoyi Gu, Mai H. Philipsen, Andrew G. Ewing

**Affiliations:** 1 Department of Chemistry and Molecular Biology, University of Gothenburg, Kemivägen 10, 412 96 Gothenburg, Sweden; chaoyi.gu@chem.gu.se; 2 Department of Chemistry and Chemical Engineering, Chalmers University of Technology, Kemivägen 10, 412 96 Gothenburg, Sweden; hmai@chalmers.se

**Keywords:** lipid alteration, mass spectrometry imaging, repetitive stimuli, exocytosis, plasticity

## Abstract

The mechanism of synaptic plasticity and its link to memory formation are of interest, yet relatively obscure, especially the initial chemical change in the cell membrane following transmitter release. To understand the chemical mechanism of plasticity, we studied how repetitive stimuli regulate certain membrane lipid species to enhance exocytotic release using mass spectrometric imaging. We found that increasing high-curvature lipid species and decreasing low-curvature lipids in the cell membrane favor the formation of a longer-lasting exocytotic fusion pore, resulting in higher release fraction for individual exocytotic events. The lipid changes observed following repetitive stimuli are similar to those after exposure to the cognitive enhancing drug, methylphenidate, examined in a previous study, and offer an interesting point of view regarding the link between plasticity and memory and cognition.

## 1. Introduction

Synaptic plasticity is a concept that has been considered to play an important role in the formation of memory [1]. Thus, a significant number of studies have been carried out attempting to understand the mechanisms underlying plasticity. The duration of synaptic plasticity can be either short-term or long-term, ranging from the time scale of milliseconds or minutes to hours or even a lifetime [2,3]. By applying high-frequency repeated stimuli, activity-dependent plasticity is induced at the presynaptic cell and observed as a change of the strength of synaptic transmission [2,4]. This change of the presynaptic cell can be observed as either an augmentation of transmission, resulting in an increased amount of transmitter release as well as a higher possibility of exocytotic release in response to stimulation, or a depression, which is partly caused by the depletion of the readily-releasable pool of vesicles [5,6]. Since large dense-core vesicles (LDCVs) show short-term activity-dependent plasticity, cell models possessing LDCVs have frequently been used for studies focusing on the mechanism of activity-dependent plasticity [7,8,9]. The presence of LDCVs in pheochromocytoma (PC12) cells, a dopaminergic cell line established in 1976, and the ability to undergo calcium-dependent exocytosis motivate the use of this cell model to study activity-dependent plasticity [10].

We have previously studied the effect of activity-dependent plasticity, which is induced by repetitive stimuli, on several cell efficacies related to neurotransmission strength in PC12 cells and found that repetitive stimuli regulate the stability of the exocytotic fusion pore to adjust the release fraction [11]. Exocytosis is a critical and tightly regulated process employed by neurons to convey signals. The initiation of exocytosis involves the merging of vesicular and cellular membranes to form a transient fusion pore, and the rearrangement of the phospholipid structure of the two bilayer membranes is essential for this fusion process [12,13]. Additionally, the expansion of the fusion pore requires the incorporation of lipids [14]. Therefore, it is of interest to understand how phospholipids in the cellular membrane are changed in response to repetitive stimuli in order to promote the stabilization of the fusion pore.

Time-of-flight secondary ion mass spectrometry (ToF-SIMS) imaging is a technique that enables the visualization and detection of lipid molecules in cellular membrane. It is a surface-sensitive technique and allows label-free identification as well as relative quantification of multiple biomolecules down to the subcellular scale. Thus, ToF-SIMS has become an important tool in multiple research areas, especially in the biological field [15,16]. This technique has been applied to analyze changes of lipid species in cells and tissues caused by drug treatments, diseases, or injuries [17,18,19,20,21]. Furthermore, the connection between lipid changes and the alteration of exocytotic release induced by chemical effectors or drugs has been investigated by multiple studies in PC12 cells using ToF-SIMS [22,23,24]. In this work, we used ToF-SIMS imaging to investigate a new paradigm for short-term cellular memory [11] and found that an increased amount of high-curvature lipids, together with a decreased level of low-curvature lipids, enables the formation of a more stable fusion pore. Thus, a higher fraction of neurotransmitters is released, representing a form of nearly instantaneous plasticity.

## 2. Results

### 2.1. Alteration of Lipids in the Cellular Membrane Caused by Repetitive Stimuli

ToF-SIMS imaging was performed with a TOF.SIMS 5 instrument (ION-TOF GmbH, Münster, Germany). To determine the alteration of lipid composition in the cellular membrane induced by activity-dependent plasticity, PC12 cells were chemically and repetitively stimulated for 0, 3, or 6 times using a stimulation solution of 100 mM K^+^. The duration of each stimulus was 5 s, and the time interval between every two stimuli was 2 min (more experimental detail is included in Materials and Methods), which is consistent with the procedure described in our previous electrochemical study [11].

**Figure 1:**
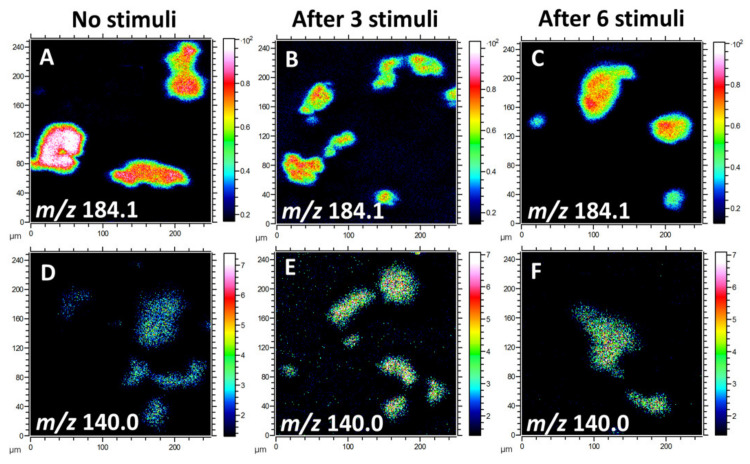
ToF-SIMS images of the lipid fragments from the plasma membranes of freeze-dried pheochromocytoma (PC12) cells with 0, 3, or 6 repetitive stimuli, showing the localization and intensities of lipid fragments in the positive and negative ion modes. (**A**–**C**) Fragment ion of phosphatidylcholine (PC) species at *m*/*z* 184.1 and (**D**–**F**) phosphatidylethanolamine (PE) fragment ion at *m*/*z* 140.0. A ToF-SIMS analysis was performed with 25 keV Bi_3_^++^ as primary ions. The total ion dose was 1 × 10^12^ ions/cm^2^.

Due to the high ion intensity of the primary ion dose and the relatively low content of lipid species in the cellular membrane, information about biomolecules at high mass range is limited to fragments. Multiple fragment ions have been identified to characterize lipid molecules in the plasma membrane. Since phosphatidylcholine (PC), phosphatidylethanolamine (PE), and phosphatidylinositol (PI) take up a high percentage of the total lipids in the cellular membrane [25], we mainly focused on the analysis of these lipid species for this study. The phosphocholine headgroup fragments, including [C_5_H_13_PNO_3_]^+^ at *m*/*z* 166.1, [C_5_H_15_PNO_4_]^+^ at *m*/*z* 184.1, and [C_8_H_19_PNO_4_]^+^ at *m*/*z* 224.1, give rise to intense ions of PC species [26]. PE has characteristic peaks at *m*/*z* 140.0 ([C_2_H_7_PNO_4_]¯) and 180.1 ([C_5_H_11_PNO_4_]ˉ). Additionally, the fragment ion [C_6_H_10_PO_8_]ˉ at *m*/*z* 241.0, representing the inositol headgroup, originates from PI species. Figure 1 illustrates the localization and intensities of fragment ions of PC and PE species on the cellular membrane after 0, 3, or 6 repetitive stimuli. In the positive ion mode, the intensity of the PC headgroup at *m*/*z* 184.1 was reduced after multiple stimuli compared to cells without any stimulation (Figure 1A–C). In contrast, in the negative ion mode, the level for the PE (*m*/*z* 140.0) fragment in the cell membrane after repetitive stimuli was higher than for the non-stimulated cells (Figure 1D–F). These findings suggest that repetitive stimuli induced a differential alteration of lipid composition in the plasma membrane.

### 2.2. Relative Levels of the Fragment Ions of Lipid Molecules

**Figure 2:**
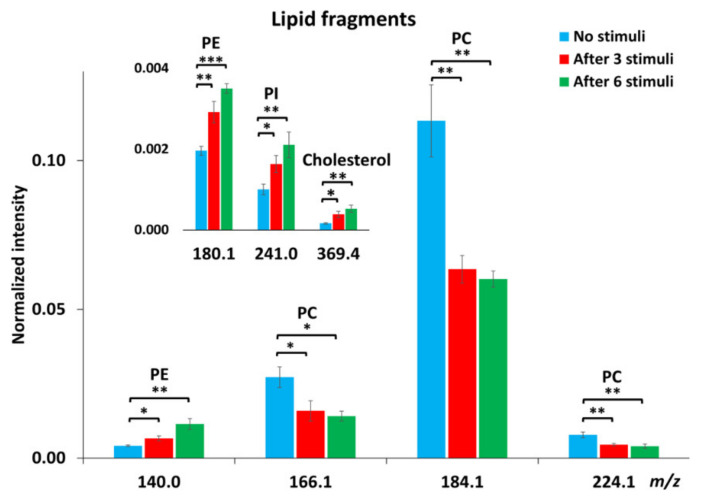
Relative intensities of certain lipid fragments, including PC, PE, phosphatidylinositol (PI) and cholesterol, in PC12 cells with no stimuli, after 3 or 6 repetitive stimuli analyzed with a Bi_3_^++^ ion source in positive and negative ion modes. The intensity of individual peaks was normalized to the number of pixels and total peak intensity. Error bars represent SEM. A student’s *t*-test was used to compare between cells with no stimuli and cells after either 3 or 6 stimuli, *: *p* < 0.05, **: *p* < 0.01, ***: *p* < 0.001, *n* = 3 for the number of experiments with three different cell generations.

To obtain more detailed information regarding lipid changes, we calculated the relative levels of the fragment ions of lipid molecules. Owing to several factors, including matrix effects and surface charging, ToF-SIMS is not an absolute quantitative method. Therefore, normalization was applied to minimize the interference of noise and the influence of sample topography on the data obtained. To do normalization, the cells were first selected as the region of interest (ROI), meaning that the spectra were characterized for the cell images only. The intensity of all single ions collected from the ROI was then normalized to the number of selected pixels and the total ion intensity of each spectrum. Figure 2 shows the alteration of lipid levels after repetitive stimuli. There was about 40% reduction in the abundance of PC fragments at *m*/*z* 166.1, 184.1, and 224.1 in the cells being repetitively stimulated. After 6 repeated stimuli, more reduction in PC levels was observed in cells compared to those after 3 stimuli, but the difference was not significant. In contrast to PC levels, repetitive stimuli elevated the intensities of PE fragments (*m*/*z* 140.0 and 180.1) and the PI fragment (*m*/*z* 241.0). More interestingly, it was observed that 6 repetitive stimuli induced approximately 40% increase in PE (*p* < 0.05, *n* = 3) and 30% increase in PI levels in comparison to the cells being stimulated only 3 times. In addition to the phospholipids, we also examined the change of cholesterol, another important component of the cellular and vesicular membranes, in response to repetitive stimuli, and the result is also included in Figure 2. The level of cholesterol in the cellular membrane increased significantly upon 3 repeated stimuli and was elevated further after 6 stimuli, showing the same trend as the alteration of PE and PI.

### 2.3. Comparison to the Amperometric Spike Data—The Prespike Foot

**Figure 3:**
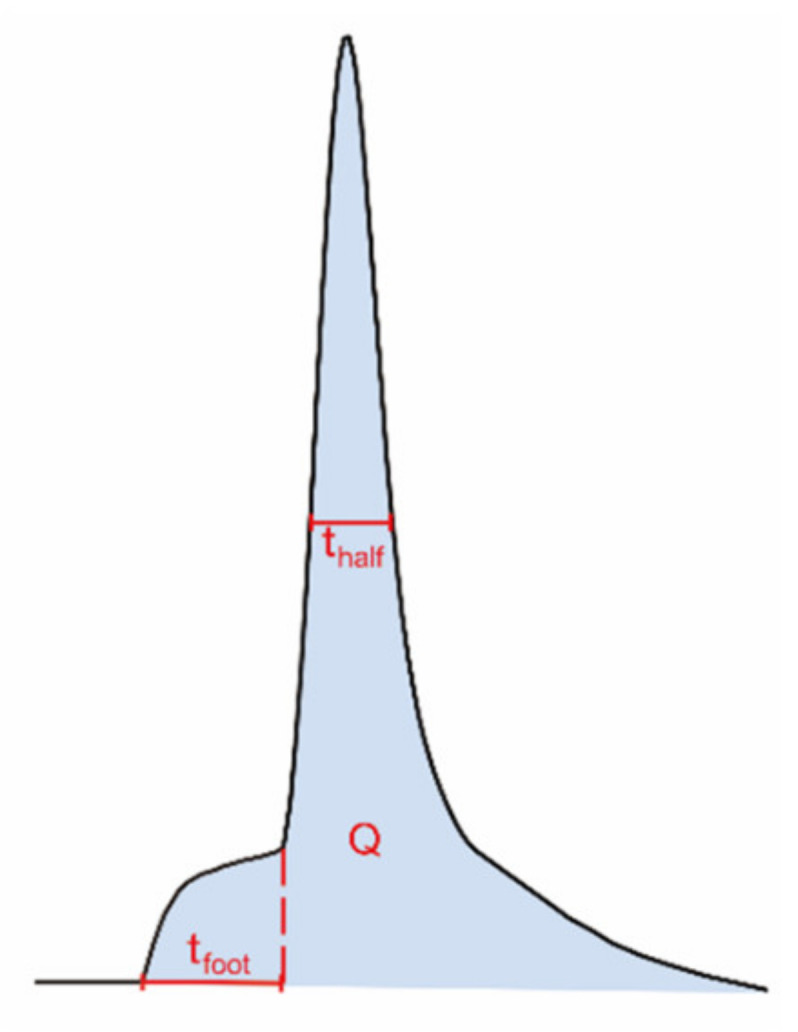
Illustration of different dynamic parameters used for exocytotic peak analysis from single-cell amperometry, including half peak width (t_half_) and foot width (t_foot_). The number of molecules (N) can be calculated using Faraday’s law, N = Q/nF; Q, time integral of the peak; *n*, number of electrons being transferred in the oxidation reaction (2 electrons for oxidation of catecholamines); F, Faraday constant.

Using electrochemical techniques, we have previously investigated how the amount of neurotransmitter release, the exocytotic fusion pore, as well as the transmitter content inside individual vesicles are regulated by repetitive stimuli [11]. The fusion pore that opens between the vesicle and cell membranes to initiate an exocytotic event is often stalled before opening further, giving a small current plateau prior to the larger exocytotic spike (Figure 3). This current results from messenger molecules passing out from the vesicle and is called the exocytotic foot [27]. This allows us to measure both the fusion pore dynamics and the broader exocytotic event and compare after repeated stimuli.

The summarized electrochemical results from our previous study are shown in Table 1 [11]. Briefly, exocytotic release is gradually enhanced upon 6 repetitive stimuli. The amounts of released transmitters are significantly higher during the third and sixth stimuli compared to the first. In contrast, the total amounts of transmitter inside single vesicles are significantly depleted upon 3 or 6 repetitive stimuli, resulting in an increased release fraction (the amount of release over the amount of storage). The vesicular storage is reduced to a higher degree after 6 stimuli than after 3 stimuli, but not significantly. The lifetime of the exocytotic fusion pore, indicated by the value t_half_ in Figure 3, becomes longer after the cells are stimulated for 3 or 6 times, demonstrating a stimulation-dependent stabilization of the fusion pore. In addition, the lifetime of pre-spike foot, represented by tf_oot_ in Figure 3, shows a trend similar to that of the t_half_ and increases with 6 repetitive stimuli as well.

## 3. Discussion

The stimulation-dependent decreases of PC and simultaneous increases of PE and PI levels are likely to facilitate, in some way, the stabilization of the fusion pore during exocytosis. Recently, many studies have suggested that exocytosis, particularly for release from LDCVs, can occur via a mode of partial or “subquantal” release instead of full release, meaning that fused vesicles are able to release only a fraction of the transmitter molecules stored in the vesicle, followed by closure of the fusion pore and a rapid endocytotic process [28,29,30,31,32,33,34,35]. The concept of partial release opens the probability for the cell to adjust the vesicular transmitter load, in addition to exocytotic release, and offers a versatile pathway for a biological system to respond to the rapid change of the surrounding environment.

The composition of membrane lipids can obviously play an important role in exocytosis. During exocytosis, lipids are relocated to form high curvature regions that are essential for membrane fusion. Knowledge of lipid organization in the plasma membrane is required to understand this biological process, and the molecular shapes of different lipid molecules are likely important factors influencing the rate of exocytosis [36]. The shapes of lipids are dependent on the relative sizes of their headgroups and acyl chains [37]. PC, which has a cylindrical shape, forms flat monolayer structures with low curvature. PE, owing to its small headgroup to acyl chain ratio, has a conical shape and thus generates a negative curvature. Cholesterol, which has been suggested to promote membrane fusion, also has an intrinsic negative curvature [38]. Conversely, a positive curvature is generated by an inverted cone-shaped lipid, such as PI, due to its relatively larger headgroup size compared to acyl chain. Therefore, it is understandable that during repeated stimulation, in order to promote the stabilization of the fusion pore, the lipid composition of the exocytotic active zones in the cellular membrane needs to be reorganized toward a structure with increased curvature [39]. With the support of the increased amount of conical-shape high-curvature lipids, PE and PI, as well as cholesterol at the fusion sites in the membrane, the fusion pore can be open for a longer period of time, allowing for an increased fraction of molecules to be released.

**Figure 4:**
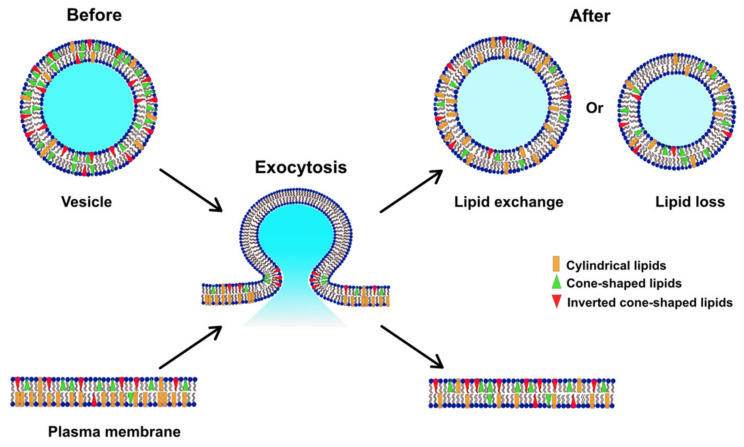
Model illustrating how the lipid composition of the plasma membrane might be altered during exocytosis induced by repetitive stimuli.

In Figure 4, we suggest a model for the alteration of the lipid composition in the plasma membrane following repetitive stimuli. Before stimulated exocytosis, the cellular membrane has a higher percentage of PCs compared to PEs and PIs, representing a relatively low curvature [25]. Vesicles, on the other hand, consist of a higher amount of total PEs and PIs and lower PC content in comparison [40,41]. During repeated stimulation, lipids at exocytotic release sites can redistribute to assist in stabilizing the fusion pore, consistent with the dynamics observed for exocytosis and with the exocytotic foot analysis in Table 1. Our data show that the plasma membrane, after repetitive stimuli, possesses a decreased level of PC and increased levels of PE and PI, which might be due to lipid exchange with vesicles during exocytosis, or a loss of certain lipids (especially PC) to form new vesicles or other membranous organelles.

Studies of Alzheimer’s disease revealed that the memory loss observed in Alzheimer’s disease patients is associated with decreased levels of PE and PI in the hippocampus [42]. Moreover, a study in *Drosophila melanogaster* showed that cocaine causes an elevation of PC abundance and a depletion of PE and PI levels [43]. Conversely, methylphenidate (MPH) appears to decrease PC levels and increase the amounts of PE and PI, which resembles the effect of repetitive stimuli observed in this work. In comparison, the lipid alteration induced by either memory loss in Alzheimer’s disease or cocaine contrasts with the lipid changes observed following repetitive stimuli or MPH. Both cocaine and MPH are psychostimulants targeting dopaminergic transmission, but they have opposite effects on cognitive performance. Many studies have supported a positive effect of MPH on various cognitive abilities, including memory, attention, and executive function [44]. In contrast, cocaine impairs cognitive function, primarily memory [45]. Recently, Zhu et al. showed that cocaine and MPH have different effects on exocytotic release fraction in PC12 cells [46]. Cocaine reduces the fraction whereas MPH enhances it. Again, the effect of MPH on release fraction is similar to what is observed with repetitive stimuli. These data suggest that the alteration of certain lipid species—to be specific, decreases in the level of PC and increases in the abundance of PE and PI—might be an important factor in the mechanism associating synaptic plasticity with memory and cognition. It is plausible that changes in lipid composition following stimulated exocytosis are a starting point for short-term memory formation.

## 4. Materials and Methods

### 4.1. Chemicals and Solutions

Chemicals were purchased from the Sigma-Aldrich company. Otherwise, their origin is stated. All chemicals were of analytical grade. The composition of the isotonic solution was 150 mM NaCl, 5 mM KCl, 1.2 mM MgCl_2_, 2 mM CaCl_2_, 5 mM glucose, and 10 mM HEPES. The 100 mM K^+^ stimulation solution was 55 mM NaCl, 100 mM KCl, 1.2 mM MgCl_2_, 2 mM CaCl_2_, 5 mM glucose, and 10 mM HEPES. A 150 mM ammonium formate solution in water was used for washing samples prior to ToF-SIMS analysis. All solutions were adjusted to pH 7.4 with 3M NaOH. Before the experiments, the solutions were filtered with a vacuum filtration device.

### 4.2. Cell Culture

PC12 cells were provided by Lloyd Greene at Columbia University and grown on cell culture flasks coated with type IV collagen (Corning Biocoat, Fisher Scientific, Sweden). The complete growth medium for the cells included 85% RPMI-1640 medium, 5% fetal bovine serum, and 10% donor equine serum, and the culture was maintained at 37 °C in an incubator with 7% CO_2_ and 100% humidity. The propagation of the cells was done once confluence was reached, typically every 7–9 days, and the medium was changed every 2–3 days.

### 4.3. Sample Preparation for ToF-SIMS Experiments

For ToF-SIMS experiments, PC12 cells were sub-cultured on silicon wafers coated with poly-d-lysine. After growing for 3 days, the cell medium was removed, and the cells were washed with pre-warmed isotonic solution and bathed in isotonic solution during stimulation. The cells were then stimulated with 100 mM K^+^ stimulation solution 0, 3, or 6 times. The duration of each stimulus was 5 s, and the resting time between every two stimuli was 2 min. 2 min after the stimuli, the cells were washed in a warm solution of the volatile salt ammonium formate to remove the cell medium and salts ions. The samples were then frozen in liquid nitrogen and subsequently freeze-dried for the ToF-SIMS analysis.

### 4.4. ToF-SIMS Imaging

The analysis of the cell samples was performed in a TOF.SIMS 5 instrument (ION-TOF GmbH, Münster, Germany) using a 25 keV bismuth primary ion beam to raster several regions of 250 × 250 µm^2^. The ToF-SIMS images were collected in high mass resolution mode with a mass resolution of m/∆m 5000 at *m*/*z* 500. Images were acquired with 256 × 256 pixels, resulting in a beam size of approximately 1 µm. The pulsed primary ion current of the Bi_3_^++^ ion was 0.3 pA and the maximum primary ion dose density was 1 × 10^12^ ions/cm^2^. The charging on the sample surface was compensated using low energy electron flooding.

### 4.5. Data Analysis

All the spectra and images were processed and analyzed using the SurfaceLab 6 software (version 6.3 ION-TOF, GmbH). The signals for [CH_3_]^+^, [C_2_H_5_]^+^, [C_5_H_12_N]^+^, and [C_5_H_15_PNO_4_]^+^ were used to calibrate the mass spectra obtained from the positive ion mode. In the negative ion mode, the mass spectra were calibrated using peaks including [CH_2_]ˉ, [CH_3_]ˉ, [CN]ˉ, and [C_16_H_32_O_2_]ˉ. The cells were defined as regions of interest (ROI) to minimize the background interference. To calculate the relative abundance, the intensities of all single peaks were normalized to the number of selected pixels of the ROI and total ion intensity.

## 5. Conclusions

The effect of repetitive stimuli on lipid composition in cellular membranes was investigated using ToF-SIMS imaging. Lipids that generate a high curvature in the membrane, including PE, PI, and cholesterol, have increased abundance after repetitive stimuli, and lipid species like PC, contributing to a low curvature in the membrane, show a decrease. These changes in abundance are likely important factors in stabilizing the fusion pore during activity-induced exocytotic plasticity. The lipid changes caused by repetitive stimuli suggest a possible mechanism relating synaptic plasticity to the formation of memory.

## Figures and Tables

**Table 1:** Summary of vesicular content, exocytotic release, t_half_, and t_foot_ for 6 repetitive stimuli ^1^ [11].

	N (10^4^) ^2^	t_half_^3^ (ms)	t_foot_^4^ (ms)
Vesicular content (no stimuli)	16.07 ± 1.26	x	x
Exocytosis during 1st stimulus	9.10 ± 0.72	1.47 ± 0.14	2.03 ± 0.13
Exocytosis during 2nd stimulus	10.10 ± 1.01	1.64 ± 0.19	2.38 ± 0.15
Exocytosis during 3rd stimulus	10.73 ± 0.94	1.57 ± 0.20	2.13 ± 0.13
Vesicular content (after 3 stimuli)	13.11 ± 0.90	x	x
Exocytosis during 4th stimulus	10.64 ± 1.20	1.66 ± 0.16	2.16 ± 0.15
Exocytosis during 5th stimulus	12.14 ± 2.08	1.78 ± 0.20	2.43 ± 0.18
Exocytosis during 6th stimulus	11.89 ± 1.48	1.82 ± 0.14	2.82 ± 0.28
Vesicular content (after 6 stimuli)	12.04 ± 0.87	x	x

^1^ The data for N and t_half_ are shown as means of medians for each cell ± SEM. The data for t_foot_ are shown as mean ± SEM. ^2^ Number of molecules released during exocytosis or amount of transmitters being stored in individual vesicles. ^3^ Width of exocytotic peak at its half amplitude (Figure 3), represents the duration of the exocytotic fusion pore. ^4^ Width of exocytotic pre-spike foot (Figure 3), represents the duration of the foot.

## Abbreviations

LDCVs	large dense-core vesicles
PC12	pheochromocytoma
ToF-SIMS	Time-of-flight secondary ion mass spectrometry
PC	phosphatidylcholine
PE	phosphatidylethanolamine
PI	phosphatidylinositol
MPH	methylphenidate
ROI	Regions of interest
